# Description of a new species of the *Charaeacoomani* group (Coleoptera: Chrysomelidae: Galerucinae) from Vietnam with a key to species

**DOI:** 10.3897/BDJ.9.e72158

**Published:** 2021-09-23

**Authors:** Dinh T Nguyen, Loan Thi Ho, Son Giang Nguyen

**Affiliations:** 1 Institute of Ecology and Biological Resources, Vietnam Academy of Science and Technology, Ha Noi, Vietnam Institute of Ecology and Biological Resources, Vietnam Academy of Science and Technology Ha Noi Vietnam

**Keywords:** Asia, biodiversity, barcoding, islands, taxonomy

## Abstract

**Background:**

The genus *Charaea* Baly is distributed in the eastern Palaearctic, Himalayas, China and adjacent countries of the Oriental Region. Currently, 59 species of the genus *Charaea* have been recorded. The species of *Charaea* is characterised with a robust tubular aedeagus that terminates with a more or less distinct apical process with the *Charaeacoomani* group having an internal sac with long sharp lateral sclerites. Up to now, 13 species of this group have been described in the Oriental Region, four of which are found in Vietnam.

**New information:**

*Charaeadinhcuongi***sp. nov.** is described as a new species, based on specimens collected from Phu Quoc Island in southern Vietnam. Colour photographs of habitus and body details and DNA barcode sequences are presented. An identification key is provided for all Vietnamese species from the *Charaeacoomani* group.

## Introduction

The genus *Charaea* (Chrysomelidae: Galerucinae) was described, based on *Charaeaflaviventris* Baly, 1878 (Baly 1878). The genus *Charaea* has a complicated taxonomic research history. Until recently, several species of the genus *Charaea* were found in various genera, such as *Taphinellina* Maulik, 1936 (Wilcox 1973, Medvedev 1998), *Calomicrus* Dillwyn, 1829 and *Exosoma* Jacoby, 1903 (Gressitt and Kimoto 1963, Kimoto 1989, Kimoto 2004). Just in the past several years, they were cumulated to the genus *Charaea* (Beenen and Warchałowski 2010, Bezděk 2012, Bezděk 2015, Bezděk 2017, Bezděk and Lee 2014, Bezděk and Viswajyothi 2019). Biology and host plants of the genus *Charaea* are unknown. Types of behaviour of the adults are observed to be evidently floricolous in most of Taiwanese species and several specimens were also collected after dark with lights (Bezděk and Lee 2014).

The species *Charaeacoomani* (Gressitt & Kimoto, 1963) was originally described under the name *Calomicruscoomani* from Hoa Binh Province, northern Vietnam by Gressitt and Kimoto (1963), then renamed under the name *Taphinellinacoomani* in Wilcox (1973) and Beenen (2010) until Beenen and Warchałowski (2010) corrected the name to *Charaeacoomani*. The *Charaeacoomani* group was tentatively proposed by Bezděk (2012), Bezděk and Lee (2014), Bezděk (2015), based on the specific structure of the aedeagus and, particularly, the internal sac with long sharp lateral sclerites. Currently, the *Charaeacoomani* group comprises 13 species distributed throughout the Oriental Region and the identification keys for this group were offered by Bezděk (2017) and Romantsov (2020). Up to now, four species are recorded in Vietnam: *C.bezdeki* Romantsov, 2020, *C.coomani* (Gressitt & Kimoto, 1963), *C.kelloggi* (Gressitt & Kimoto, 1963) and *C.khanhoanica* Romantsov, 2018 (Gressitt and Kimoto 1963, Kimoto 1989, Bezděk 2017, Romantsov 2018, Romantsov 2020). In the present study, a new species of the *Charaeacoomani* group is described from Phu Quoc Island, Kien Giang Province, southern Vietnam and an identification key for all Vietnamese species from *Charaeacoomani* group is provided.

## Materials and methods

The specimens were collected using the beating method in the tree canopy in the tropical forests of Phu Quoc National Park, Kien Giang Province, southern Vietnam and transferred immediately to vials containing 96% ethanol.

Photographs were taken with a Nikon Ds – Fi3 camera mounted on a Nikon SMZ800N stereomicroscope and processed with NIS – Element imaging software. Images of the same objects at different focal planes were combined using the Helicon Focus 7 software.

DNA was extracted from the whole identified specimen using the QIAamp DNA Investigator (QIAGEN) kit following the manufacturer’s protocol. Primers LepF1 (forward direction) (5’-ATTCAACCAATCATAAAGATATTGG-3’) and LepR1 (Reverse direction) (5’-TAAACTTCTGGATGTCCAAAAAATCA-3’) (Hebert et al. 2004) were used to amplify a 658 base pair (bp) fragment of the *COI* gene. Each PCR reaction mixture contained 2.5 µl of 10x reaction buffer (Evrogen, Russia), 0.5 µl of 10 mM dNTPs, 0.5 µl of 10 µM forward primer, 0.5 µl of 10 µM reverse primer, 1 µl of 25 mM Mg^2+^, 2 µl of template DNA, 0.2 µl of thermostable Taq DNA polymerase (Evrogen, Russia) and 17.8 µl deionised water. The PCR protocol used is as follows: initial denaturation at 94°C for 3 mins; 35 cycles of denaturation at 94°C for 30 s, annealing at 42°C for 40 s, elongation at 72°C for 60 s; and final elongation at 72°C for 5 mins. PCR products were visualised via electrophoresis using a 1.5% agarose gel and then purified using ammonium acetate and cold isopropanol. They were sequenced in both directions using the BigDye Terminator v.3.1 Cycle Sequencing kit (Applied Biosystems, Foster City CA, USA) with the same PCR primers. Specimens after DNA extraction were mounted dry and labelled with a voucher number for future reference at the Institute of Ecology and Biological Resources (IEBR). Forward and reverse Sanger sequences were assembled in a consensus sequence (Geneious Prime 2019.0.4) and then submitted to the Barcoding of Life Database (BOLD; www.boldsystems.com) with the BIN: BOLD: AEH1826; and Genbank (https://www.ncbi.nlm.nih.gov/genbank) with the accession number MW407948.1.

## Taxon treatments

### Charaea dinhcuongi

Nguyen, 2021 sp. n.

88C837F3-BC76-53B2-A4D4-BE78CEB06B95

urn:lsid:zoobank.org:act:5C4BCFA9-836C-461B-AA8E-46AA93723274

MW407948.1

#### Materials

- **Type status:**Holotype. **Occurrence:** occurrenceDetails: http://www.boldsystems.org/index.php/API_Public/specimen?bin=BOLD:AEH1826; catalogNumber: IEBR; recordedBy: Dinh T. Nguyen, Trinh Dinh Cuong; individualID: Gal_81_2019; individualCount: 4; sex: 3 Males, 1 female; lifeStage: adult; preparations: whole animal; disposition: in collection of author; associatedSequences: Genbank: MW407948.1; **Taxon:** scientificName: *Charaeadinhcuongi* Nguyen, 2021; phylum: Arthropoda; class: Insecta; order: Coleoptera; family: Chrysomelidae; genus: Charaea; **Location:** locationID: Ba Tan moutain, Phu Quoc district; higherGeographyID: Phu Quoc Islands, Kien Giang province; higherGeography: Vietnam, Kien giang Province,; continent: Asia; island: Phu Quoc; country: Vietnam; countryCode: VN; stateProvince: Kien Giang; verbatimElevation: 101 m a.s.l.; verbatimLatitude: 10.24105 N; verbatimLongitude: 103.96967 E; verbatimCoordinateSystem: decimal degrees; **Event:** samplingProtocol: beating; samplingEffort: beating tree canopy in the paths in forest in 8 hours/day in two weeks/ time * 2times, 50 km by motobike; year: 2019; month: July; day: 9; habitat: forest; **Record Level:** type: life specimens; institutionID: IEBR; institutionCode: Institute of Ecology and Biological Resources; collectionCode: NAFOST.106.09-2019; basisOfRecord: livingSpecimen

#### Description

Measurements. Males: 3.86–3.90 mm, female: 3.60 mm. Body oblong, convex, slightly broadened posteriorly, 1.72 times as long as wide. The dorsal side is glabrous, oval, convex. Colour bluish-black, abdomen yellowish-brown; mandibles black; labrum black; Antennae with antennomeres I–III bluish-black and IV-XI black; legs black with femora lustrous and bluish-black (Fig. 1).

Male (Fig. 2a, b): Labrum transverse, anterior margin not emarginate in middle and with four thin pale setae; in the middle of the labrum with six setae arranged in a horizontal row, lateral margins rounded and convergent. Clypeus transverse and small, anterior margin of clypeus straight with few long setae, impunctate. Frontal ridge large, convex, impunctate, transverse, isosceles triangular with the apex in the middle of two antennal callis. The frontal tubercles separated from the vertex by a distinct transverse furrow. Eyes are large, strongly convex. Interantennal space is twice as wide as the transverse diameter of the antennal socket and interocular space is twice as wide as the transverse diameter of the eye. Antennae is robust, slightly longer than half body length, length ratio of antennomeres equals: 100: 53: 73: 105: 105: 105: 105: 116:116: 137 9.5: 5: 7: 10: 10: 10: 10: 10: 11: 11: 13, antennomeres I–III lustrous, covered with sparse setae, antennomeres IV-XI dull, covered with dense short setae.

Pronotum (Fig. 2c) is strongly convex, oval, without any discal impressions, 1.32 times as wide as long, broadest in middle. Disc of pronotum, covered with two kinds of punctures, sparse fine punctures cover the whole surface, sparse larger punctures are cumulated in two wide longitudinal stripes. Anterior margin concave, posterior margin convex, lateral margins rounded. Anterior margin unbordered, posterior margin thinly bordered, lateral borders distinctly wider; anterior angles swollen, posterior angles obtusangulate. All angles with large setigerous pore-bearing long seta, additional short setae visible on lateral margins in anterior half.

Scutellum glabrous, bluish-black, impunctate and triangular.

Elytra 1.36 times as long as wide, widest at near apex; elytral disc glabrous and densely covered with moderately large confused punctures. Humeral calli well developed. Epipleura strongly broadened at base, gradually narrower to middle and disappearing at apical third.

Legs (Figs 1b, d, 2e, f) slender. All tibiae with an apical spur in both sexes. Tarsomeres I elongate, subparallel, tarsomeres II subtriangular. Ventral sides of pro-and mesotarsomeres I with large sensilla patch (Fig. 12). Length ratio of protarsomeres and mesotarsomeres I–IV equals 10: 6: 5: 11, protarsomere and mesotarsomere I 2.1 times as long as wide. Length ratio of metatarsomeres I–IV equals 12: 7: 6: 14, metatarsomere I 3.0 times as long as wide. Claws appendiculate. Anterior coxal cavities open posteriorly. Prosternal process very narrow, not visible between procoxae. Mesosternum free, not covered by a process of metasternum. Head ventrally, pro- and mesoventrite lustrous, glabrous and impunctate. Mesepisterna, mesepimera, metaventrite,and metepisterna covered with dense short setae.

Abdomen (Figs 1b, d, 2d) yellow, covered with dense short setae with five distinctly visible ventrites; hind margins of first to third ventrite straight and fourth ventrite concave; last ventrite trilobed, middle lobe with straight cut apex, its surface slightly impressed throughout. Pygidium convex with round apex.

Aedeagus (Fig. 3a, b, c) length 1.74 mm, with apex forming apical process, two lateral sides of the process narrow in the middle, apex with small emargination in middle and lateral view is straight. Internal sac with two lateral sclerites, long, convergent and sharp apices (Fig. 3d, e).

Female similar to male (Fig. 1c, d), but ventral sides of pro– and mesotarsomeres I without sensilla patch. Posterior margin of the last ventrite triangular. Pygidium with round apex. Spermatheca with nodulus distinctly wider than cornu, cornu C-shaped, proximal spermathecal duct almost straight as in Fig. 4a. Sternite VIII pentagonal, tignum slender, 1.5 times longer than sternite VIII as in Fig. 4b.

#### Diagnosis

*Charaeadinhcuongi***sp. nov.** is similar to the species having a long apical process of aedeagus (*C.kelloggi* (Gressitt & Kimoto, 1963), *C.latha* Bezděk, 2017 and *C.mimicum* (Medvedev, 1998)). In *Charaeadinhcuongi* **sp. nov.**, the apical process of the aedeagus with two lateral sides narrow in the middle, apex with small emargination in the middle, in the lateral view the process is straight. In *C.kelloggi*, the apical process of the aedeagus is convergent, with apex narrow and rounded. In *C.latha*, the apical process of the aedeagus is wide with apex transversely cut and, in lateral view, the process is widely round and bent up. In *C.mimicum*, the apical process of the aedeagus is longer with the apex truncate.

The shape of internal sclerites (long with sharp apices) in *Charaeadinhcuongi* **sp. nov.** is very similar in to *C.kelloggi* (Gressitt & Kimoto, 1963), *C.latha* Bezděk, 2017, *C.coomani* (Gressitt & Kimoto, 1963) and *C.mimicum* (Medvedev, 1998). The species *C.coomani* has a subtriangular aedeagus apex.

#### Etymology

Dedicated to Trinh Dinh Cuong, who helped me collect specimens.

#### Distribution

Phu Quoc Island, Kien Giang Province, southern Vietnam.

## Identification Keys

### Key to all Vietnames species from the *Charaeacoomani* group

**Table:** 

1	The apex of the aedeagus is subtriangular or forms an apical process	2
–	The apex of the aedeagus with the apical orifice	4
2	The apex of the aedeagus is subtriangular. Internal sclerites in aedeagus parallel, without spines (Fig. 5b)	*C.coomani* (Gressitt et Kimoto, 1963)
–	The apex of the aedeagus forms an apical process	3
3	The apical process of the aedeagus is narrow, convergent, with rounded tip (Fig. 5c)	*C.kelloggi* (Gressitt et Kimoto, 1963)
–	The apical process of the aedeagus is wider, two lateral sides of the process narrower in the middle and apex with small emargination in middle (Fig. 3)	* C. dinhcuongi * **sp. nov.**
4	The anterior margin of the aedeagus with very deep triangular emargination (Fig. 5d)	*C.khanhoanica* Romantsov, 2018
–	The anterior margin of the aedeagus is slightly convex with small emargination in middle (Fig. 5a)	*C.bezdeki* Romantsov, 2020

## Supplementary Material

XML Treatment for Charaea dinhcuongi

## Figures and Tables

**Figure 1a.**
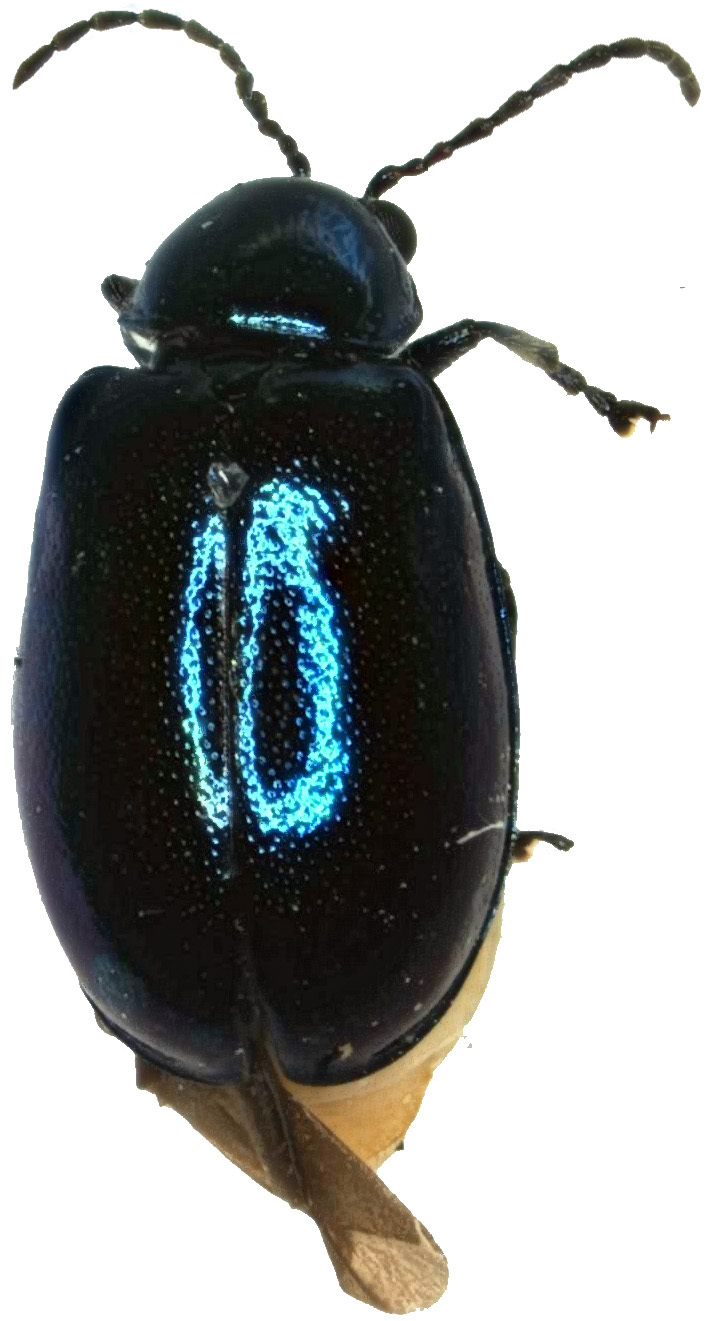
male, dorsal view (holotype)

**Figure 1b.**
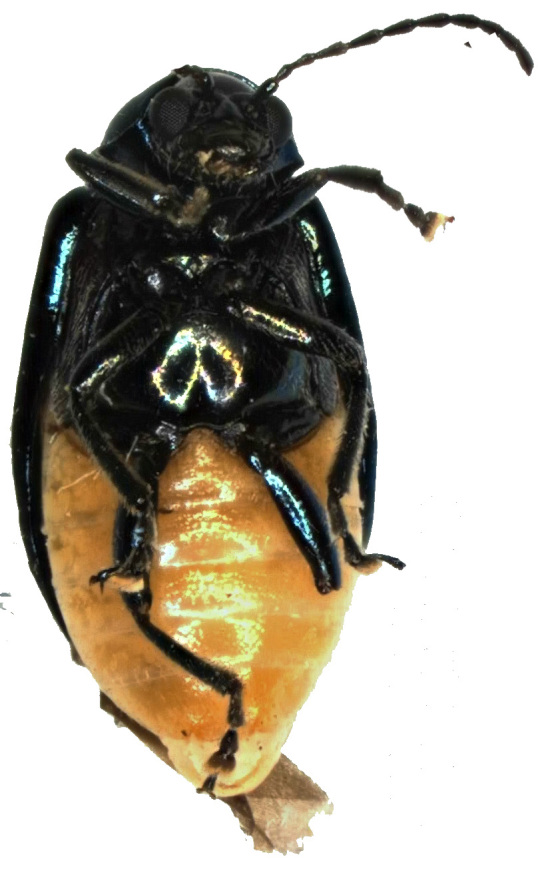
male, abdomen view

**Figure 1c.**
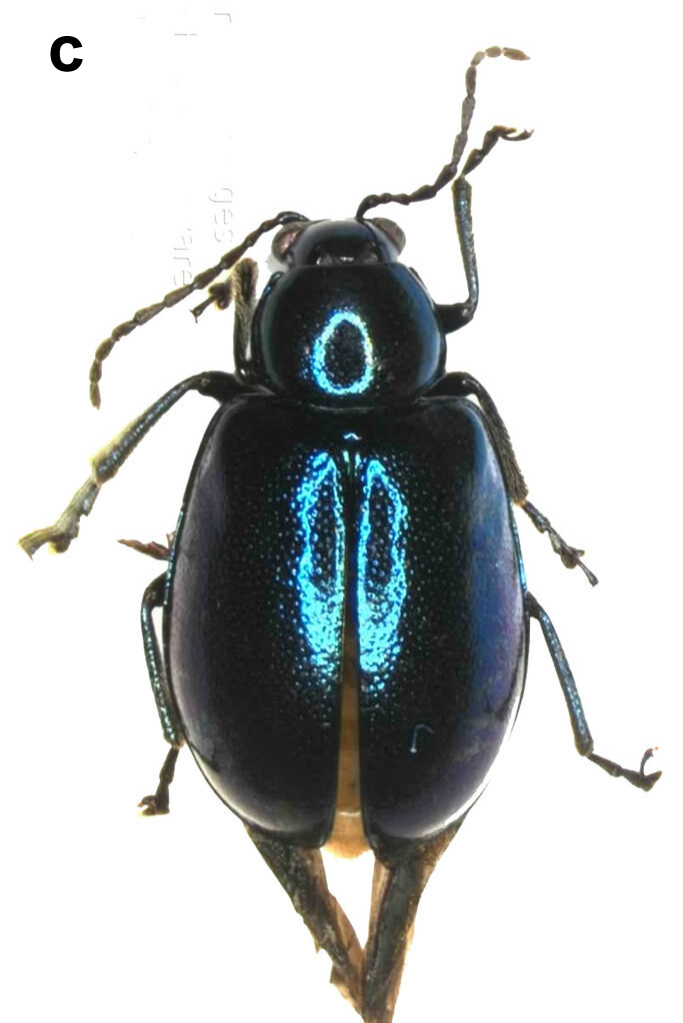
female, dorsal view

**Figure 1d.**
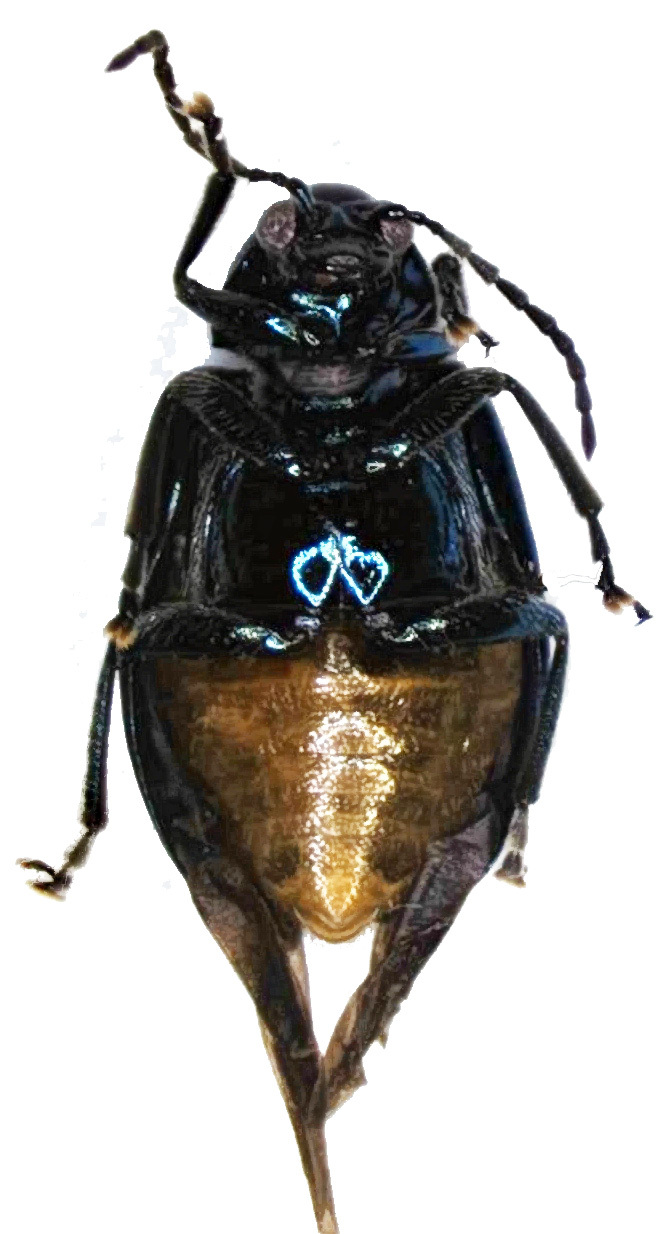
female, abdomen.

**Figure 2a.**
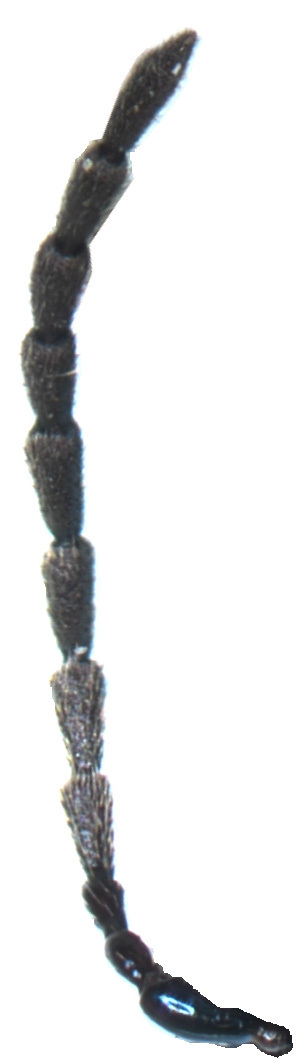
male antenna

**Figure 2b.**
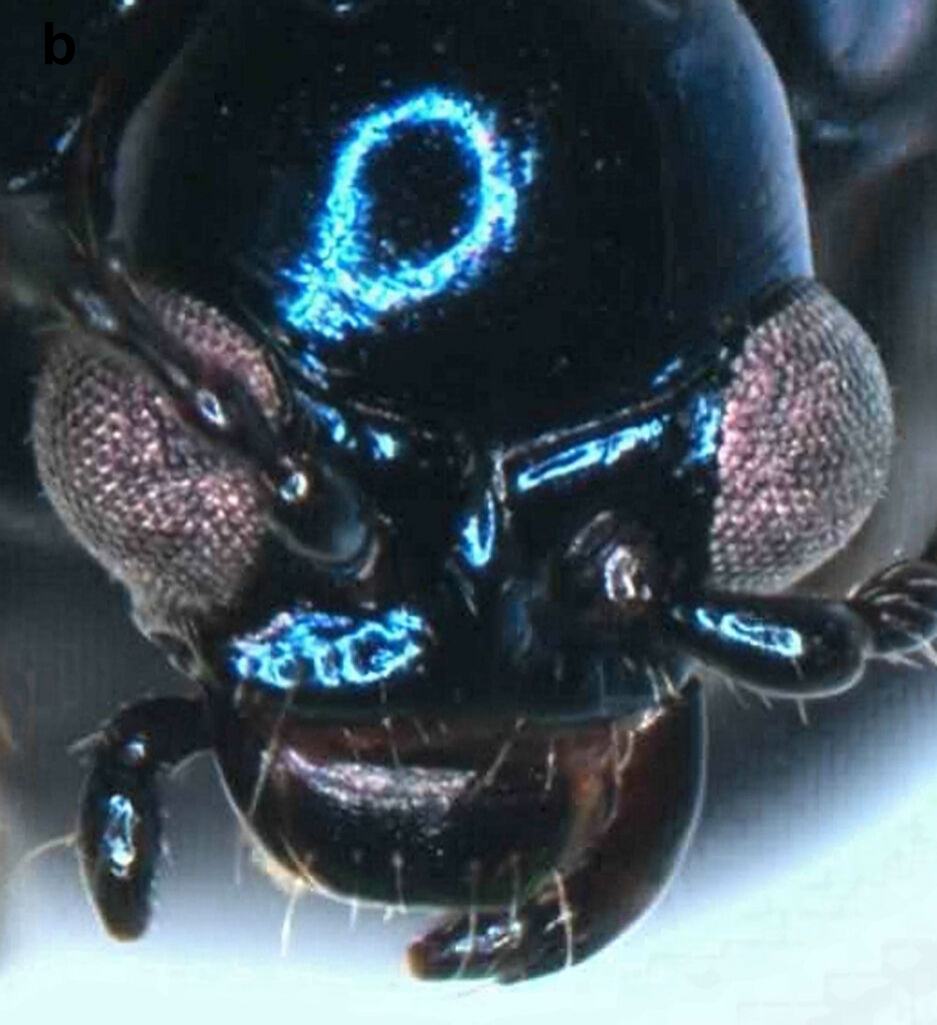
male head, frontal view

**Figure 2c.**
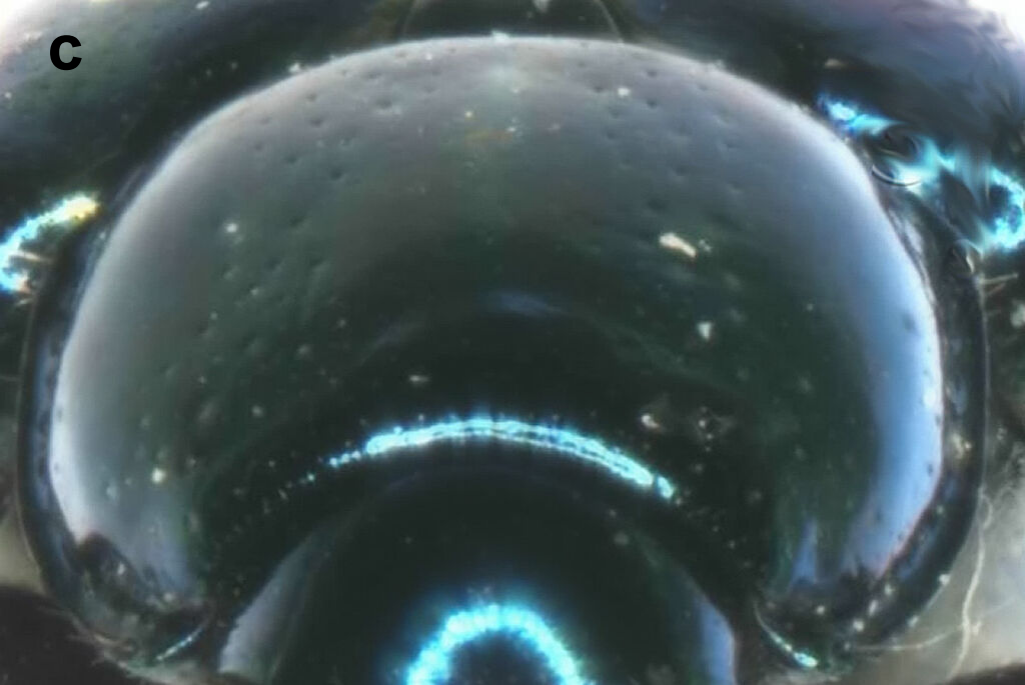
male pronotum view

**Figure 2d.**
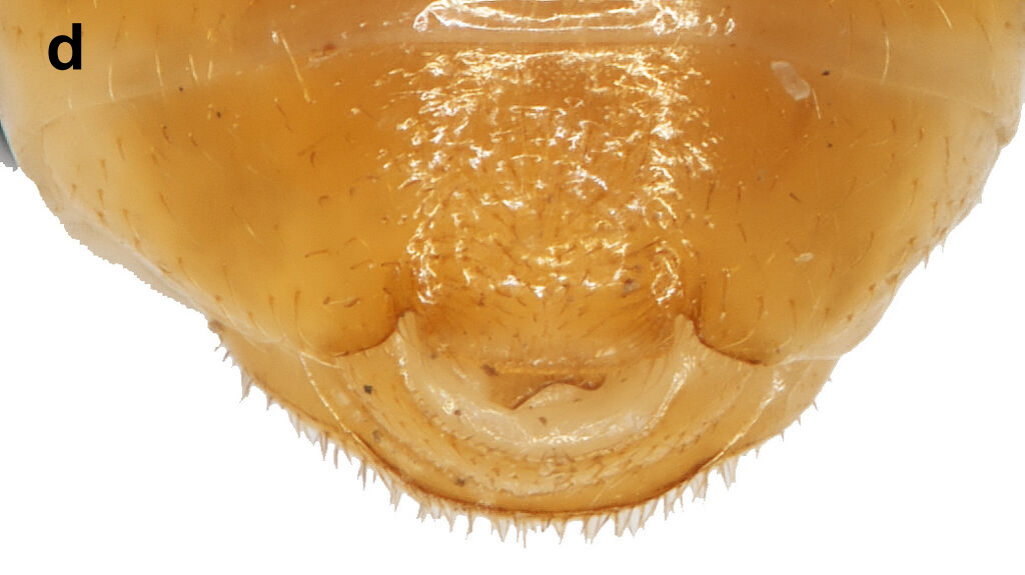
last male abdominal

**Figure 2e.**
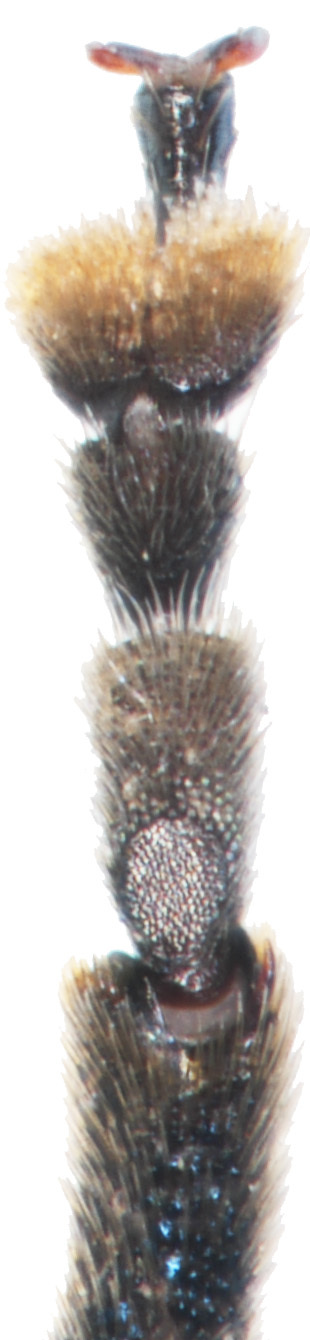
male protarsus

**Figure 2f.**
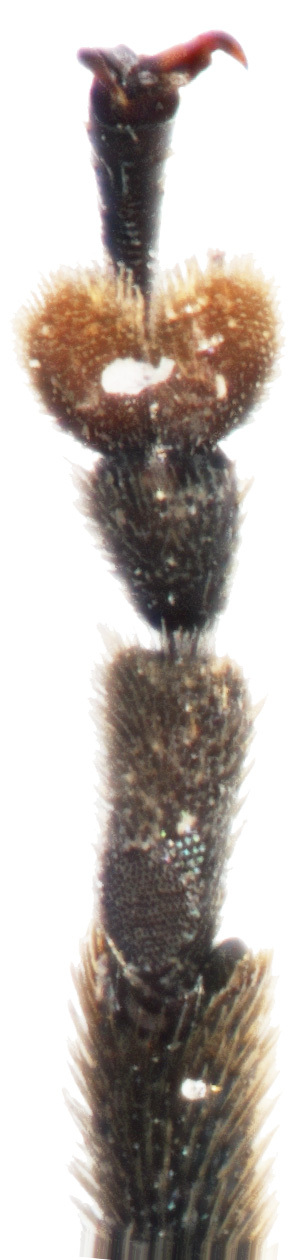
male mesotarsus.

**Figure 3a.**
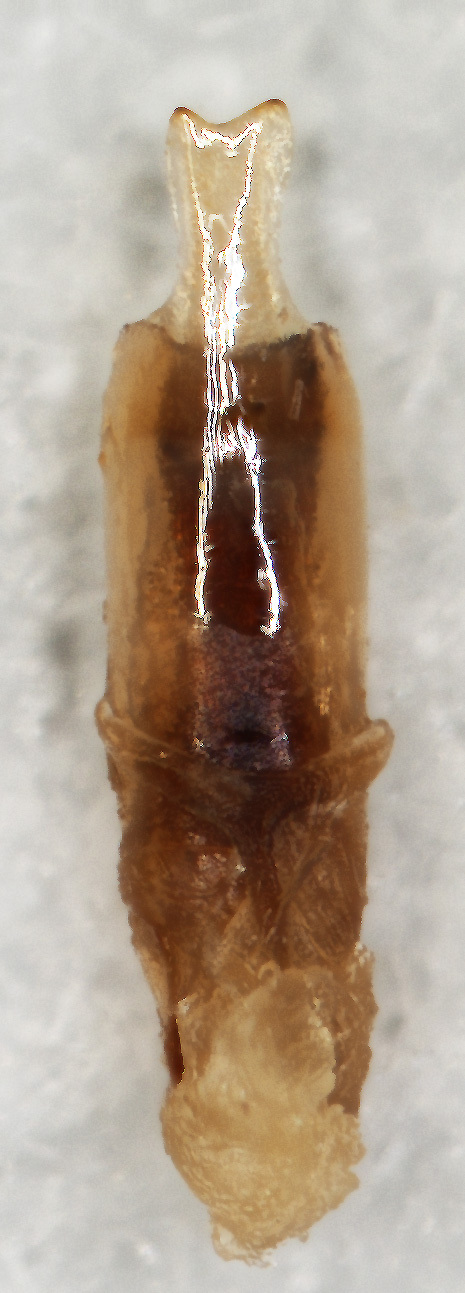
aedeagus, dorsal view

**Figure 3b.**
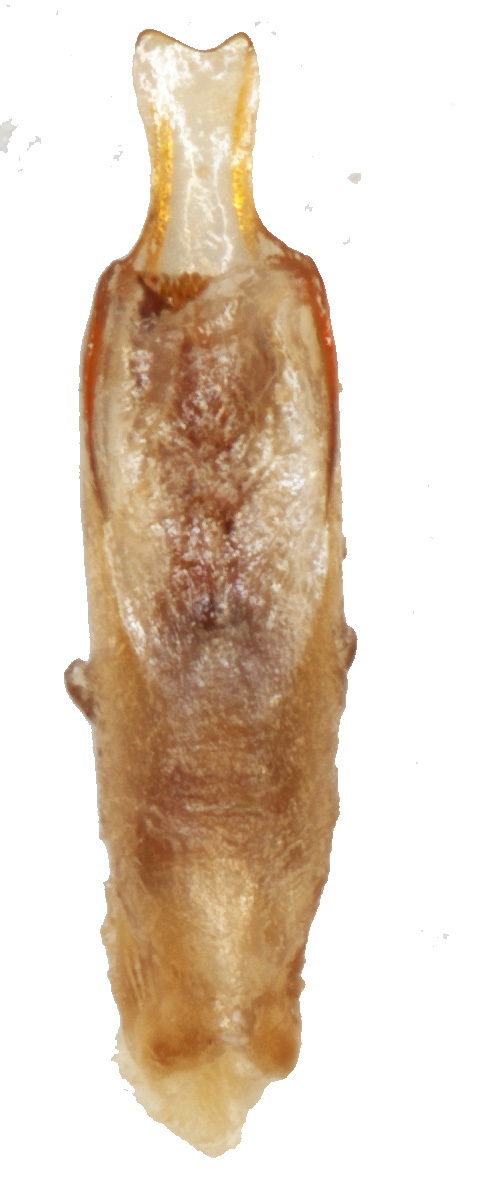
aedeagus, ventral view

**Figure 3c.**
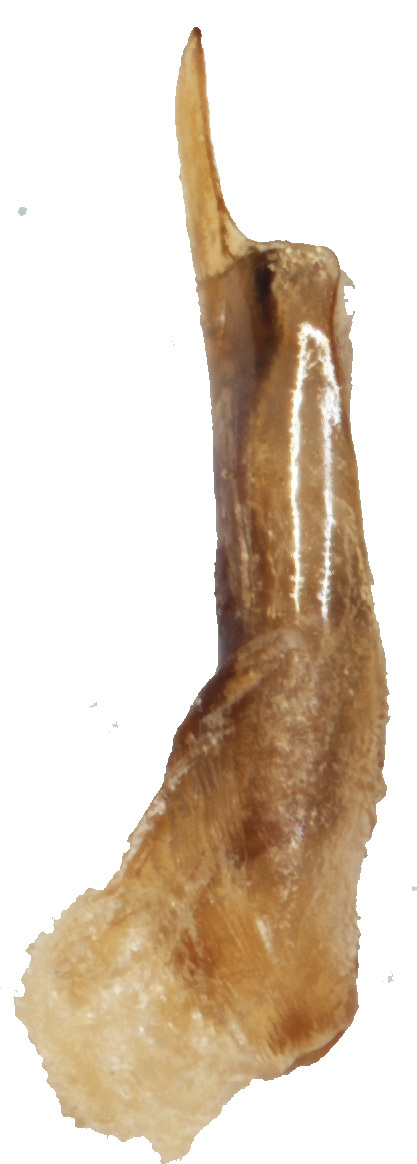
aedeagus, lateral view

**Figure 3d.**
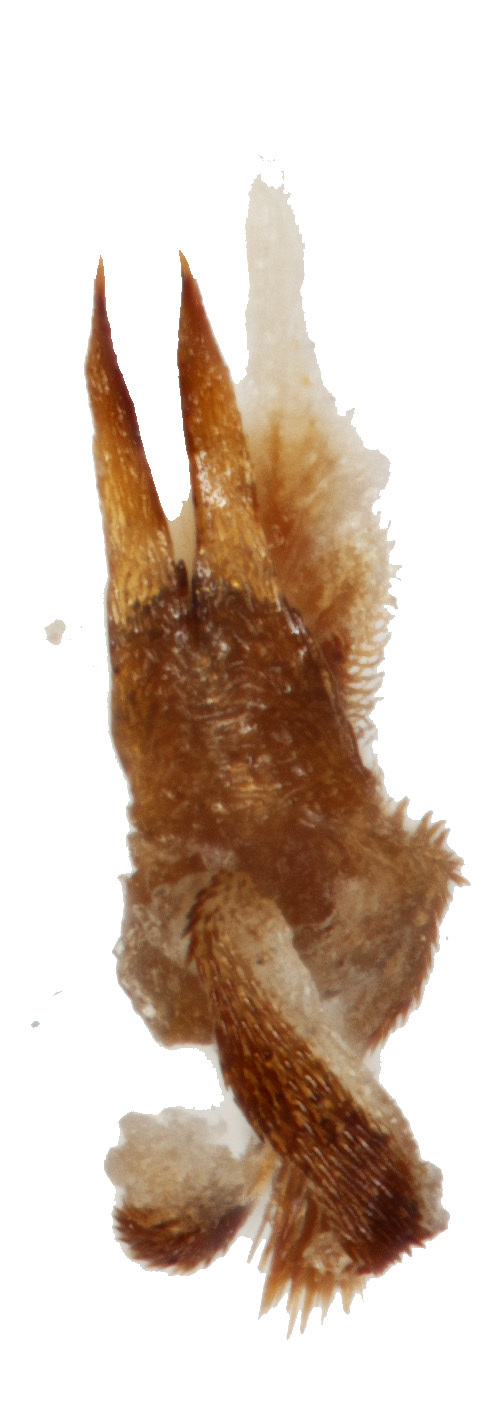
internal sclerite

**Figure 3e.**
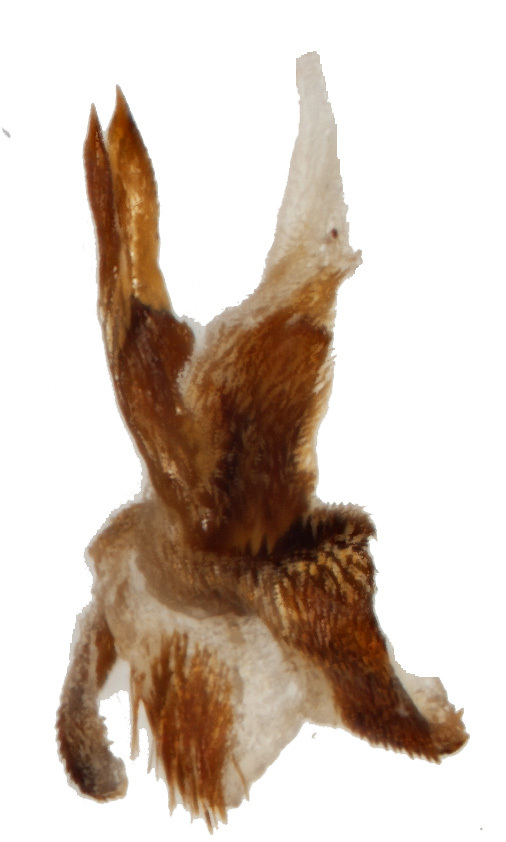
internal sclerite.

**Figure 4a.**
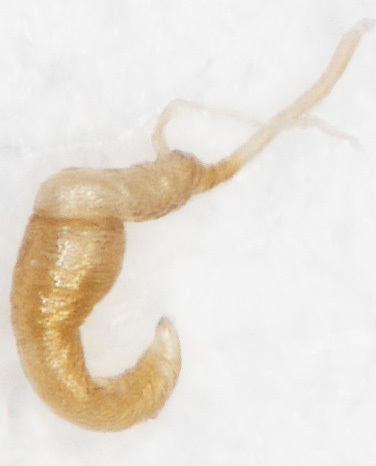
spermatheca

**Figure 4b.**
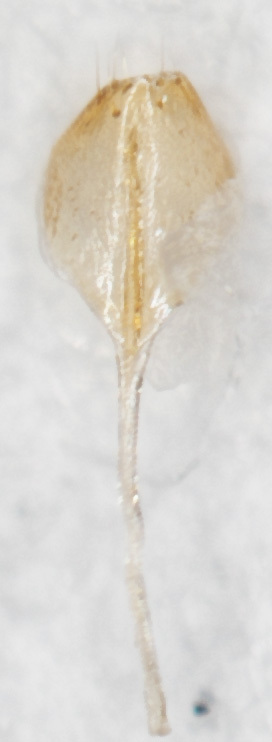
sternite VIII.

**Figure 5a.**
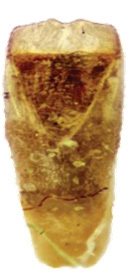
*C.bezdeki* (from Romantsov 2020)

**Figure 5b.**
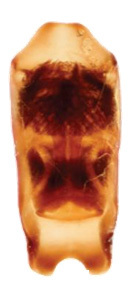
*C.coomani* (from Bezděk 2017)

**Figure 5c.**
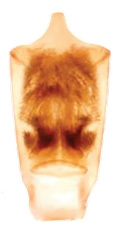
*C.kelloggi* (from Bezděk 2017)

**Figure 5d.**
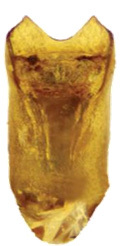
*C.khanhoanica* (from Romantsov 2020).

## References

[B6872272] Baly J. S. (1878). Descriptions of the phytophagous Coleoptera collected by the late Dr. F. Stoliczka during Forsyth’s expeditionto Kashgar in 1873–74. Cistula Entomologica.

[B7341591] Beenen R., Löbl I., Smetana A. (2010). Catalogue of Palaearctic Coleoptera. Chrysomeloidea..

[B6872489] Beenen R., Warchałowski A. (2010). *Charaeapseudominutum* n. sp., an undescribed but not unknown galerucine beetle (Coleoptera, Chrysomelidae, Galerucinae). Entomologische Blätter für Biologie und Systematik der Käfer.

[B6872525] Bezděk J. (2012). Taxonomic and faunistic notes on Oriental and Palaearctic Galerucinae and Cryptocephalinae (Coleoptera: Chrysomelidae). Genus.

[B6872547] Bezděk J., Lee Ch. -F. (2014). Revision of *Charaea* (Coleoptera: Chrysomelidae: Galerucinae) from Taiwan. Zootaxa.

[B6872606] Bezděk J. (2015). *Charaealuzonicum* sp. nov. (Coleoptera: Chrysomelidae: Galerucinae): the first record of Charaea in the Philippines. Revue Suisse de Zoologie.

[B6872633] Bezděk J. (2017). *Charaea* Baly (1878) (Coleoptera: Chrysomelidae: Galerucinae) of Vietnam, Laos, Thailand, Myanmar and Peninsular Malaysia. Journal of Asia-Pacific Entomology.

[B7276726] Bezděk Jan, Viswajyothi Keezhpattillam (2019). Revision of *Charaea* (Coleoptera: Chrysomelidae: Galerucinae) of South India. Annales Zoologici.

[B6872650] Gressitt J. L., Kimoto S. (1963). The Chrysomelidae (Coleopt.) of China and Korea, part 2. Pacific Insects Monograph.

[B6877889] Hebert P. D.N., Penton E. H., Burns J. M., Janzen D. H., Hallwachs W. (2004). Ten species in one: DNA barcoding reveals cryptic species in the neotropical skipper butterfly *Astraptesfulgerator*. Proceedings of the National Academy of Sciences of the United States of America.

[B7338803] Kimoto S. (1989). Chrysomelidae (Coleoptera) of Thailand, Cambodia, Laos, and Vietnam IV. Galerucinae. Esakia.

[B6872462] Kimoto S. (2004). New or little known Chrysomelidae (Coleoptera) from Nepal, Bhutan and the northern territories of Indian subcontinent. Bulletin of the Kitakyushu Museum of Natural History and Human History.

[B6872453] Medvedev L. N. (1998). New Chrysomelidae (Coleoptera) from Southeast Asia in the Hungarian Natural Museum. Annales Historico-Naturales Musei Nationalis Hungarici.

[B6872668] Romantsov P. V. (2018). New species of the subfamily Galerucinae (Coleoptera: Chrysomelidae) from South-East Asia. Caucasian Entomological Bulletin.

[B6872685] Romantsov P. V. (2020). Two new species and new records of Galerucinae (Coleoptera: Chrysomelidae) from Vietnam. Caucasian Entomological Bulletin.

[B6872379] Wilcox J. A., Wilcox J. A. (1973). Coleopterorum Catalogus Supplementa.

